# Supplementary material for: Glucocorticoid Receptor Regulates and Interacts with LEDGF/p75 to Promote Docetaxel Resistance in Prostate Cancer Cells
Source: Cells. 2023 Aug 11;12(16):2046. doi: 10.3390/cells12162046 (PMC10453226; doi:10.3390/cells12162046)
Supplement: Supplementary file 1 [file cells-12-02046-s001.zip › cells-2493203-supplementary.pdf]

Figure S1

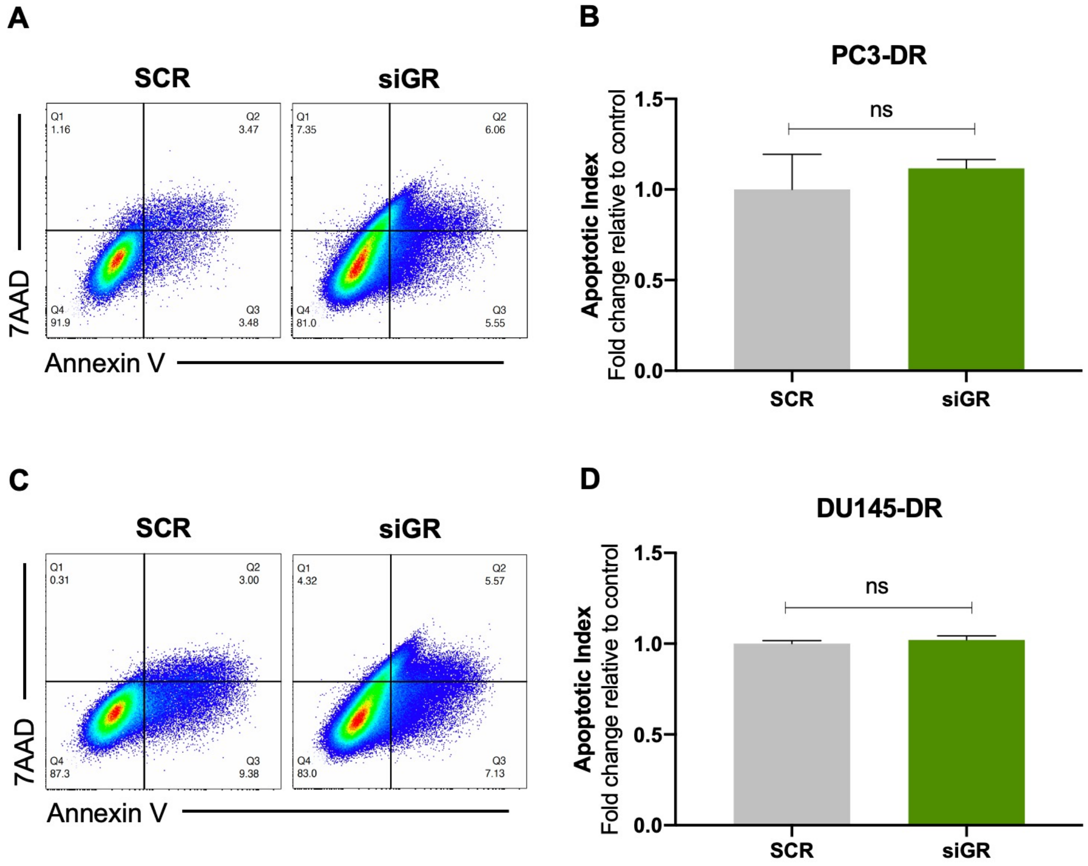

Figure S1. Transient GR knockdown does not affect PCa cell survival. siRNA mediated silencing of GR in PC3-DR (**A**), and DU145-DR (**C**) demonstrates no cytotoxic effect when comparing SCR negative control to siGR samples. SCR and siGR samples were stained with Annexin V/7AAD to assess cell death through flow cytometry. Representative plots from 3 independent experiments show the apoptotic index (**B** and **D**). Statistical analyses were performed using unpaired *t* tests comparing SCR to siGR samples. ns = not significant. Error bars represent mean +/− SEM from 3 independent experiments for each cell line.

Figure S2

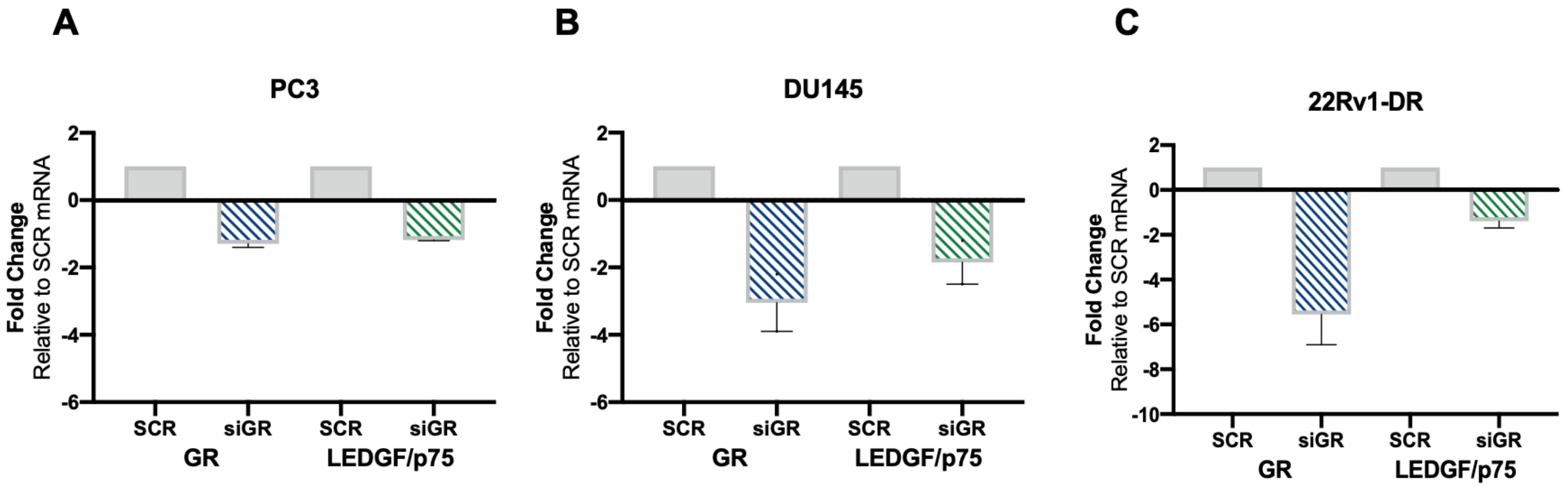

Figure S2. Transient GR knockdown decreases both GR and LEDGF/p75 mRNA levels in PCa cells. siRNA-mediated GR silencing was performed in PC3 (**A**), DU145 (**B**), and 22Rv1-DR (**C**) cells for 72h. Total RNA was extracted from PC3, DU145, and 22Rv1-DR cells after siRNA transfections. Gray bars represent SCR negative control, blue bars confirm the GR silencing transcript levels, and green bars show the decreased LEDGF/p75 transcript levels. Data was normalized to SCR negative control mRNA levels. Results represent 2 independent qPCR experiments using three biological replicates per experiment. Error bars represent mean +/− SEM.

Figure S3

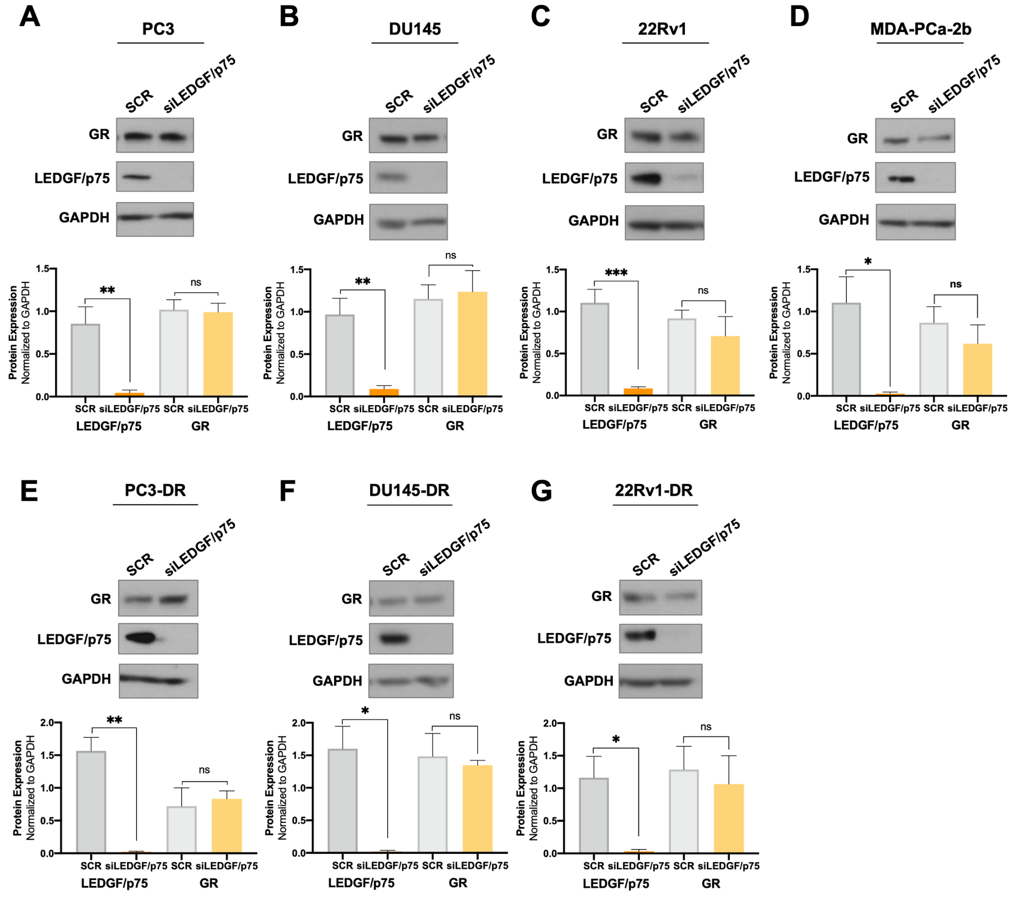

Figure S3. LEDGF/p75 silencing has no effect on GR protein expression levels in PCa cells. DTX-sensitive PC3 (**A**), DU145 (**B**), 22Rv1 (**C**), and MDA-PCa-2b (**D**) cell lines were transfected with siRNAs specific for LEDGF/p75 (siLEDGF/p75) or scrambled negative control oligos (SCR) for 72 h. LEDGF/p75 silencing resulting in no change in GR protein expression was confirmed by immunoblotting. Similar results were observed in the DTX-resistant PCa cell lines PC3-DR (**E**), DU145-DR (**F**), and 22Rv1-DR (**G**). Quantified band values for GR and HRP2 were obtained with ImageJ software and plotted as relative protein expression normalized to GAPDH. Statistical analyses were performed using unpaired *t* tests comparing SCR to siGR samples. *$p < 0.05$, **$p < 0.01$. Error bars represent mean +/− SEM from at least 3 independent experiments for each cell line.

Figure S4

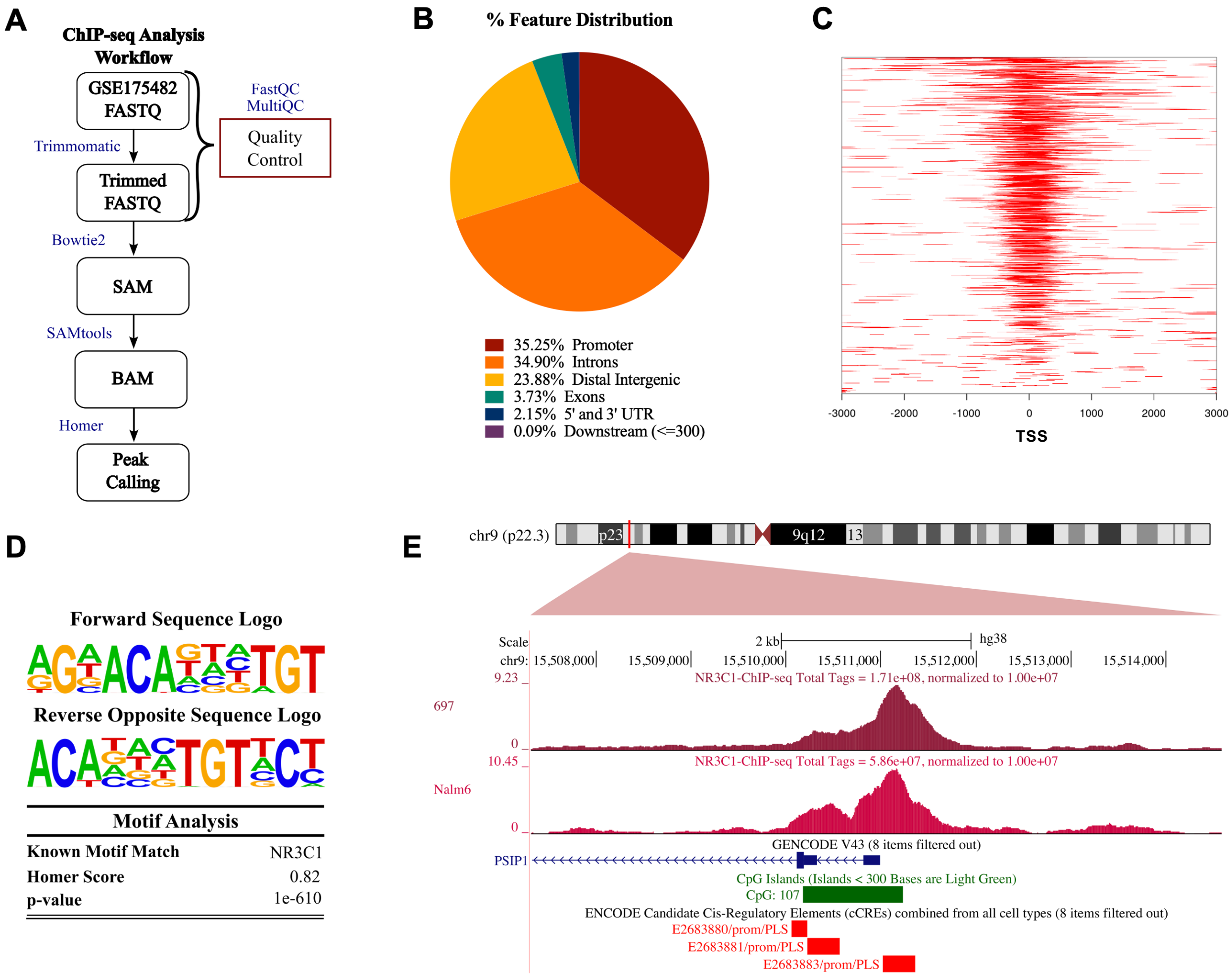

Figure S4. GR binding motifs were identified in the promoter region of the gene encoding LEDGF/p75 (*PSIP1*) using ChIP-seq in acute lymphoblastic leukemia (ALL) cell lines. Flow diagram of the analysis pipeline used for the 697 and Nalm6 ChIP samples obtained from the gene expression omnibus (GEO) database (**A**). Binding sites were distributed among multiple features, with promoters having the highest occurrence (**B**). Heatmap of ChIP binding to transcription start site (TSS) regions, zero is set as the TSS (**C**). Near the TSS of *PSIP1*, motif analysis identified a potential GR binding site (**D**). UCSC genome browser visualization of normalized peaks for *NR3C1* (gene encoding GR) near the TSS of *PSIP1*. ChIP tracks are shown in shades of burgundy, GENCODE *PSIP1* transcript is in blue, CpG islands are in green, and ENCODE promoter-like signatures are in red (**E**).

Figure S5

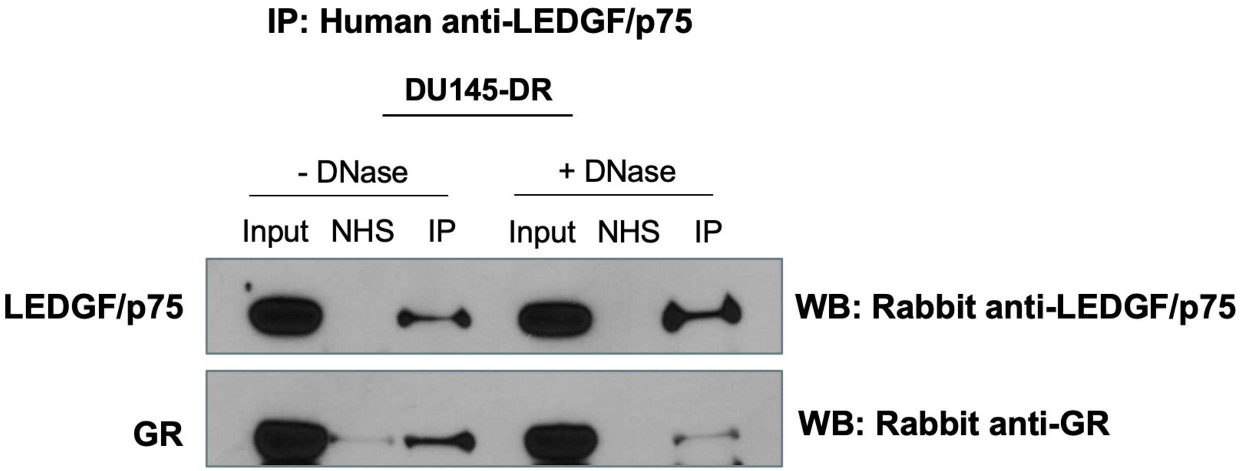

Figure S5. The endogenous interaction between LEDGF/p75 and GR interaction is not affected by DNase treatment. Immunoprecipitations were performed using specific human anti-LEDGF/p75 autoantibodies. DNase treatment of cell lysates was performed for 15 mins prior to addition of anti-LEDGF/p75 autoimmune serum or normal human serum (NHS). IP was confirmed by immunoblotting with rabbit monoclonal antibodies specific for either LEDGF/p75 or GR in DU145-DR cells. NHS was used as negative control for IP and whole cell lysates collected from IP reactions were used as input (1% of IP).

Figure S6

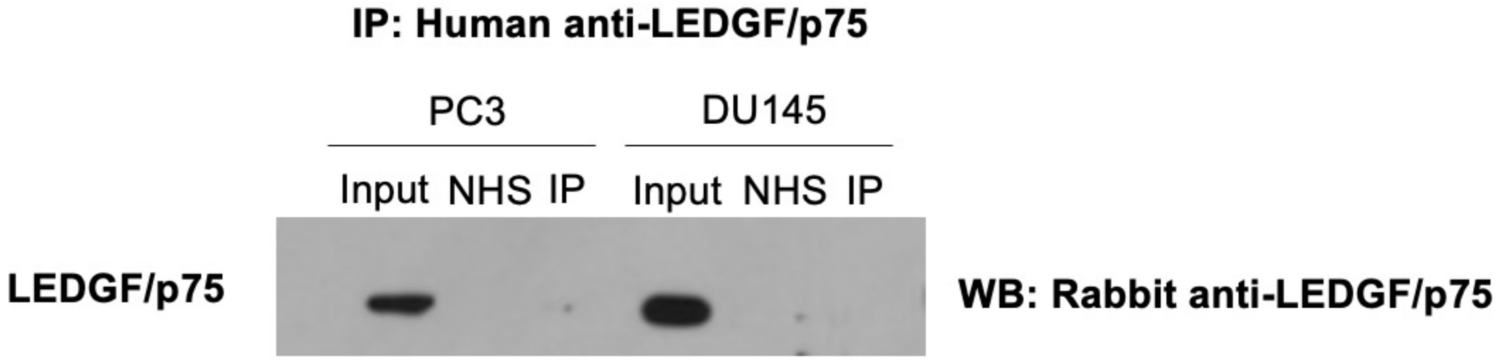

Figure S6. LEDGF/p75 immunoprecipitation is not efficient in DTX-sensitive PCa cells. Immunoprecipitations (IP) were performed using specific human anti-LEDGF/p75 autoantibodies. Normal human serum (NHS) was used as negative control for IP and whole cell lysates collected from IP reactions were used as input (1% of IP). IP confirmation was assessed by immunoblotting with rabbit monoclonal antibodies specific for LEDGF/p75 in DTX-sensitive PC3 and DU145 cells and representative immunoblots only show the immunoreactivity against LEDGF/p75 in the input (1% of IP). .

Figure S7

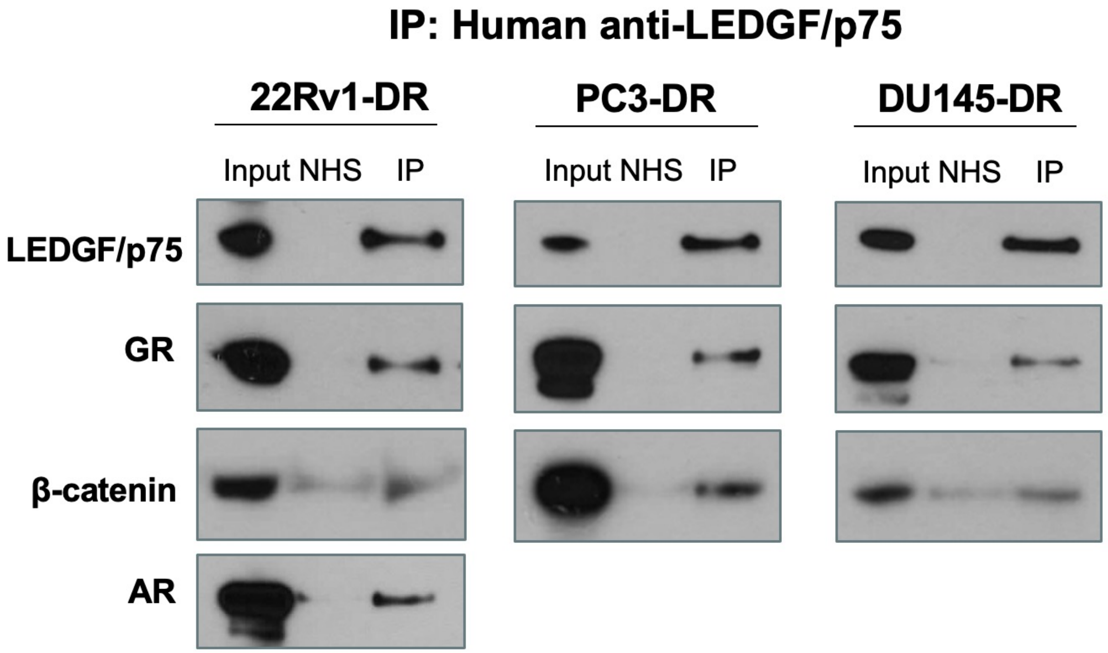

Figure S7. LEDGF/p75 and GR endogenously interact with β-catenin and AR in PCa cells. Immunoprecipitations were performed using specific human anti-LEDGF/p75 autoimmune serum. IP was confirmed by immunoblotting with rabbit monoclonal antibodies specific for either LEDGF/p75, GR, β-catenin, or AR in 22Rv1-DR, PC3-DR, or DU145-DR. Normal human serum (NHS) was used as negative control for IP and whole cell lysates collected from IP reactions were used as input (1% of IP). Independent experiments were performed at least 3 times.

Figure S8

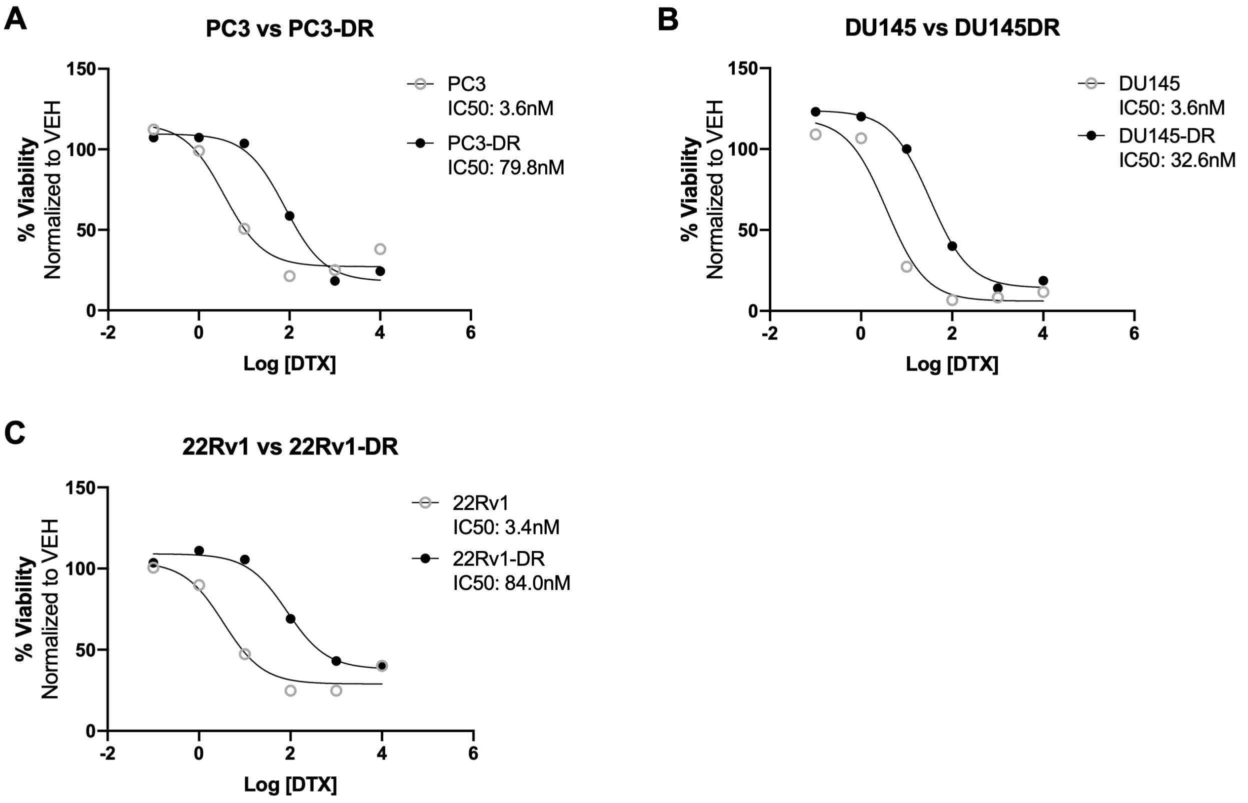

Figure S8. Validation of docetaxel-resistance (DR) in PCa cell lines. Docetaxel IC50 values, determined by MTT assays following drug treatment for 72 hours, are shown for the following parental and DR cell line pairs: (A) PC3 vs PC3-DR, (B) DU145 vs DU145-DR, and 22Rv1 vs 22Rv1-DR. DR cells were generated by continuous exposure to incrementally increasing concentrations of DTX (0.1, 1, 3, 5, 6, 7, 8, 9, 10 nM) until stable (>95%) viability was achieved at 10nM DTX. Data include at least three independent experiments and are represented as mean ± SEM.

Figure S9

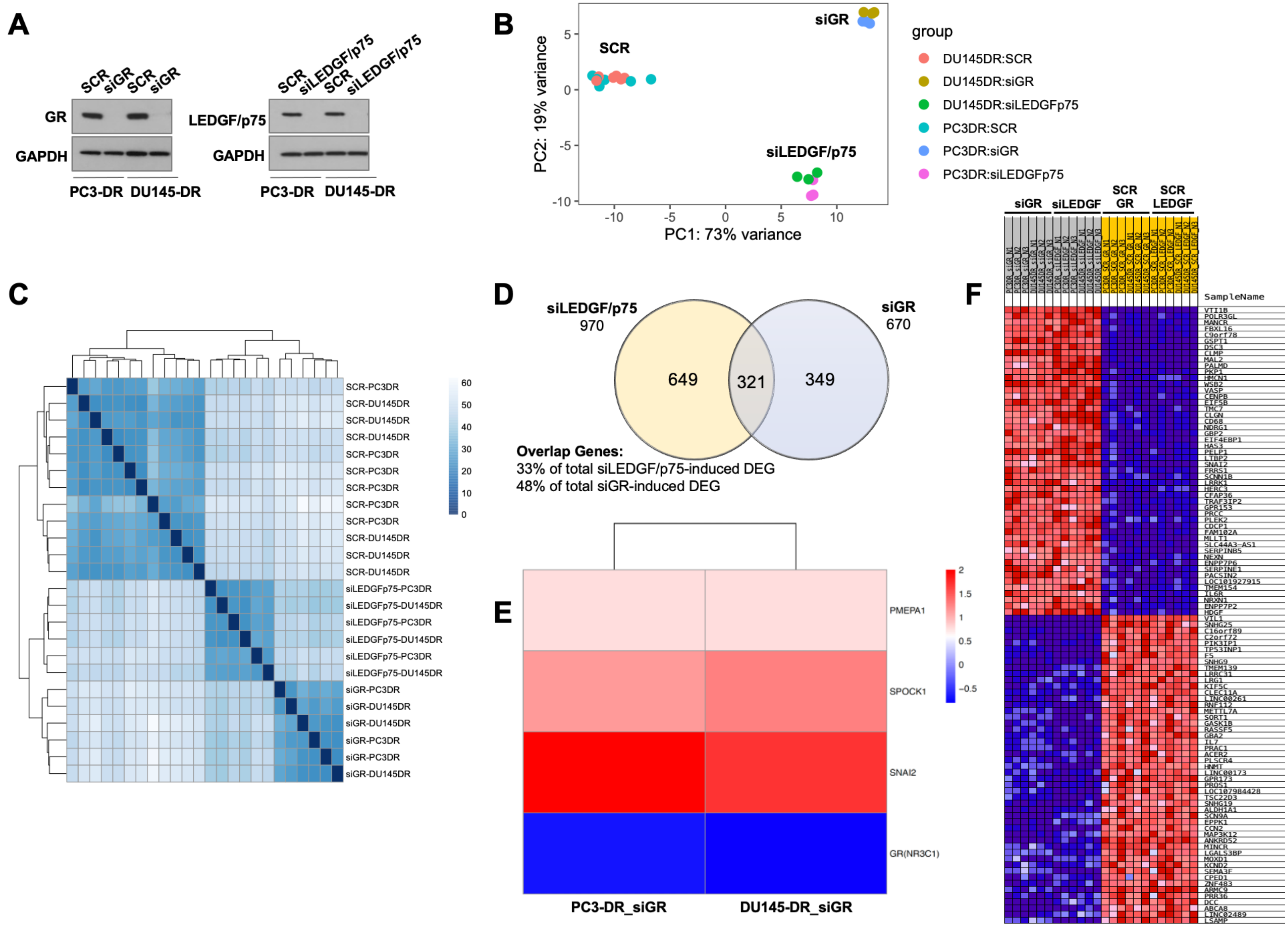

Figure S9. RNA-seq data analysis. (**A**) Representative immunoblots confirming siRNA-mediated GR or LEDGF/p75 silencing in PC3-DR and DU145-DR cells used for the RNA-seq analysis. (**B**) Principal component analysis (PCA) demonstrating the clustering of scramble (SCR), siLEDGF/p75, and siGR samples from DTX-resistant PCa cells based on gene expression profiles. (**C**) Correlation plot of SCR, siLEDGF/p75 and siGR samples from DTX-resistant PCa cell lines based on gene expression profiles. (**D**) Venn Diagram of Differentially Expressed Genes (DEGs) in siLEDGF/p75 or siGR samples from DTX-resistant PCa cell lines. (E) Heat map of *PMEPA1*, *SPOCK1*, *SNAI2* and *NR3C1* (GR) in PC3-DR and DU145-DR cells with GR silencing. (**F**) GSEA-generated heat map of the top ranked 50 overlap DEGs significantly downregulated (blue) or upregulated (red) by siGR or siLEDGF/p75, compared to SCR controls, in DTX-resistant PC3-DR and DU145-DR cells.
